# Oxidative Stress Induced by MnSOD-p53 Interaction: Pro- or Anti-Tumorigenic?

**DOI:** 10.1155/2012/101465

**Published:** 2011-10-05

**Authors:** Delira Robbins, Yunfeng Zhao

**Affiliations:** Department of Pharmacology, Toxicology & Neuroscience, Louisiana State University Health Sciences Center, Shreveport, LA 71130, USA

## Abstract

The formation of reactive oxygen species (ROS) is a result of incomplete reduction of molecular oxygen during cellular metabolism. Although ROS has been shown to act as signaling molecules, it is known that these reactive molecules can act as prooxidants causing damage to DNA, proteins, and lipids, which over time can lead to disease propagation and ultimately cell death. Thus, restoring the protective antioxidant capacity of the cell has become an important target in therapeutic intervention. In addition, a clearer understanding of the disease stage and molecular events that contribute to ROS generation during tumor promotion can lead to novel approaches to enhance target specificity in cancer progression. This paper will focus on not only the traditional routes of ROS generation, but also on new mechanisms via the tumor suppressor p53 and the interaction between p53 and MnSOD, the primary antioxidant enzyme in mitochondria. In addition, the potential consequences of the p53-MnSOD interaction have also been discussed. Lastly, we have highlighted clinical implications of targeting the p53-MnSOD interaction and discussed recent therapeutic mechanisms utilized to modulate both p53 and MnSOD as a method of tumor suppression.

## 1. Introduction

Oxidative stress has been defined as the cellular imbalance of prooxidants versus antioxidants that overwhelms the cell's capacity to scavenge the oxidative load and contributes to the pathogenesis of various diseases. Reactive oxygen species (ROS) are free radicals derived from molecular oxygen that play a key role in promoting oxidative stress. These radicals result from the incomplete reduction of oxygen mainly during mitochondrial respiration. There are several products of oxygen metabolism, both nonradicals and radicals that form ROS such as hydrogen peroxide (H_2_O_2_) and superoxide anions (O_2_^.^^−^). Contributors of ROS can modify the intracellular redox status through unfavorable interactions with endogenous regulators of oxidative stress. Superoxide radicals can interact with mitochondrial nitric oxide to form peroxynitrite which can alter antioxidant enzymes such as aconitase and the mitochondrial complexes of the electron transport chain [1]. On the other hand, the presence of oxidative stress can alter normal cellular homeostasis by modifying proteins involved in DNA repair; activating signal transduction pathways involved in cell survival and inflammation; as well as, inducing cellular apoptotic pathways that are detrimental to the cell. For many years, scientists have tried to combat free radical generation and superoxide production through the utilization of the exogenous antioxidant supplementation, such as ascorbate, vitamin E, as well as linoleic acid. However, many of these trials have failed showing no significant decrease in cancer incidence, death, or major cardiovascular events [2]. Herein, we will focus on several novel signaling pathways affecting ROS generation, such as p53 signaling and the interaction between p53 and manganese superoxide dismutase (MnSOD) and how to potentially target these pathways for cancer therapy. 

## 2. Oxidative Stress

Oxidative stress has been repeatedly shown to contribute to the progression of multiple diseases, such as cancer [3], diabetes [4], ulcerative colitis [5], cardiovascular disease [6], pulmonary disease [7] as well as neurodegenerative diseases [8]. Nevertheless, the biological significance of oxidative stress can be beneficial or detrimental depending on certain parameters such as concentration, duration of action, cell type exposed, the type of free radicals and reactive metabolites involved, and the activities of the associated signal transduction pathways.

The mitochondrial electron transport chain remains to be one of the main sources of intracellular oxidative stress [9]. During mitochondrial respiration, electrons flow through four integral membrane protein complexes to finally reduce molecular oxygen to water. However, approximately 1-2% of molecular oxygen undergoes incomplete reduction, resulting in the formation of superoxide anions and mitochondria-mediated ROS generation [10]. Though mainly produced from mitochondrial respiration, superoxide anions can be detoxified via endogenous antioxidant enzymes such as manganese superoxide dismutase (MnSOD) to hydrogen peroxide, which is further converted to water via the enzymatic actions of various antioxidant enzymes including glutathione reductases, peroxiredoxins, glutathione transferases, as well as catalase which all function in the removal of hydrogen peroxide.

Nevertheless, it is common for cells in response to stress to enhance ROS generation. Oxidoreductases are enzymes that are often activated during the cellular stress response and catalyze the transfer of electrons from the electron donor (reductant) to the electron acceptor (oxidant) [11] with associated formation of superoxide anions and ROS as byproducts. There are several enzymes that act as oxidoreductases and contribute to intracellular ROS generation, such as cyclooxygenase [12], lipoxygenase [12, 13], cytochrome P450 enzymes [14], nitric-oxide synthase [15], xanthine oxidase [16], and mitochondrial NADH: ubiquinone oxidoreductase (complex I) [17]. NAPDH oxidases of the Nox family are also oxidoreductases that produce superoxide anions as a primary product and one of the key sources of intracellular ROS formation. NADPH oxidases (Nox) are endogenous enzymatic heterogenic complexes that reduce molecular oxygen to superoxide, in conjunction with NADPH oxidation, which can be converted to various ROS. Nox can be activated by a myriad of cellular stress stimuli such as heavy metals [18, 19], organic solvents [20], UV and ionizing irradiation [21, 22]. Once the cellular stress response is initiated two cytosolic regulatory units of Nox, p47^phox^, p67^phox^, and the small G protein Rac translocate to the membrane and associate with cytochrome *b558* (consisting of two subunits gp91phox (Nox2) and p22phox), which acts as a central docking site for complex formation [23]. Emerging evidence has linked Nox enzymes to oxidative stress that may contribute to disease progression [11, 17, 24, 25]. The radicals generated from Nox activation are capable of modulating various redox-sensitive signaling pathways involved in the activation of mitogen-activated protein kinases (MAPKs) and transcription factors (NF-*κ*B) [26–28] causing regulation of Nox activation to be complex. 

Oxidative stress can be generated endogenously, as well as promoted exogenously by multiple environmental factors. Ultraviolet irradiation (UV) is an environmental promoter of oxidative stress. UV is known to damage DNA and other intracellular proteins through direct and indirect mechanisms. UV exists in three forms UVA (400–320 nm), UVB (320–290 nm), and UVC (290–100 nm). UVA and UVB are the most biologically significant, with UVC being most absorbed by ozone [29]. UV is known to directly induce the cross-linking of neighboring pyrimidines to form pyrimidine dimers in DNA that result in mutagenic DNA lesions [30–35]. However, UV is known to promote ROS generation that can damage a large number of intracellular proteins and can indirectly damage DNA.

Associated with oxidative damage is lipid peroxidation. High levels of ROS are detrimental and can cause damage to various biomolecules, which include the fatty acid side chains of membrane lipids that form reactive organic products such as malondialdehyde and 4-hydroxynonenal, both of which can generate DNA adducts and point mutations [36]. Lipid peroxidation not only affects DNA stability, but can also alter lipid membrane proteins that are involved in signal transduction pathways to promote constitutive activation and downstream cellular proliferation. Furthermore, previous studies have shown that products of lipid peroxidation served as intermediates in the activation of signaling pathways that involved phospholipase A2 and the MAPK pathway, both associated with UV-induced carcinogenesis [37–39]. 

Although there are various sources of endogenous oxidative stress, mitochondria are the major cellular organelles that contribute to intracellular ROS generation. Mitochondria consume approximately 80–90% of the cell's oxygen for ATP synthesis via oxidative phosphorylation. In the early 1920s Otto Warburg and colleagues theorized that defective oxidative phosphorylation during cancer progression caused tumor cells to undergo a metabolic shift requiring high rates of glycolysis that promoted lactate production in the presence of oxygen. This phenomenon became known as aerobic glycolysis and later coined “The Warburg Effect.” Some of the metabolic enzymes that are altered during cancer progression are involved in the mitochondrial electron transport chain [40, 41]. The electron transport chain consists of a constant flow of electrons through mitochondrial intermembrane complexes with molecular oxygen being the ultimate electron acceptor. The process of the electron transport chain is used to pump protons into the mitochondrial inner membrane creating an electrochemical gradient. The gradient that is created is coupled to ATP synthesis. However, leaking electrons contribute to the incomplete reduction of molecular oxygen, resulting in superoxide anion formation. Mitochondria are readily susceptible to oxidative damage for various reasons: (1) lack of effective base excision repair mechanisms; (2) the close proximity of mitochondrial DNA to ROS generation; (3) lack of mitochondrial DNA protective histones [42]. Therefore, alterations in mitochondrial ROS generation and protection via antioxidant expression are key in the detrimental effects of disease progression.

## 3. Manganese Superoxide Dismutase (MnSOD)

Maintaining a balance between free radicals and antioxidants is required for cellular homeostasis. However, when this balance is altered in favor of free radical generation, normal physiology is altered and the pathogenesis of disease is promoted. Antioxidants are endogenous defense mechanisms utilized by the cell to fight against fluctuations in free radical generation, which include both enzymatic and nonenzymatic contributors. Ascorbic acid (Vitamin C) and *α*-tocopherol (Vitamin E) are nonenzymatic antioxidants that have been previously shown to effectively scavenge free radicals. On the other hand, antioxidants such as glutathione peroxidase and superoxide dismutase are enzymatic antioxidants that catalyze the neutralization of free radicals into products that are nontoxic to the cell. Superoxide dismutase catalyzes the dismutation of superoxide anions leading to the formation of hydrogen peroxide and molecular oxygen. Hydrogen peroxide is further detoxified to water via catalase and other endogenous antioxidant enzymes. The superoxide dismutase family consists of metalloenzymes. Currently, there are three major superoxide dismutase enzymes within the human cell: manganese superoxide dismutase (MnSOD), copper-zinc superoxide dismutase (Cu, ZnSOD), and extracellular superoxide dismutase (ECSOD). MnSOD is localized in the mitochondrial matrix [43], Cu, ZnSOD is found primarily in the cytosol [44] and can be detected in the mitochondrial intermembrane space [45], and ECSOD is a homotetrameric glycosylated form of Cu, ZnSOD found in the extracellular space [46]. 

MnSOD is ubiquitously found in both prokaryotes and eukaryotes, and its increased activity is often associated with cytoprotection against oxidants. MnSOD can be induced by various mediators of oxidative stress such as tumor necrosis factor, lipopolysaccharide, and interleukin-1 [47]. This antioxidant enzyme is nuclear encoded by a gene localized to chromosome 6q25 [48], a region often lost in cancers such as melanoma [49]. MnSOD is synthesized in the cytosol as a larger precursor with a transit peptide on the N-terminus and imported to the mitochondrial matrix via proteolytic processing to the mature form [50]. Most cancer cells and *in vitro* transformed cell lines have diminished MnSOD activity compared to normal counterparts [51]. In addition, deficiencies in MnSOD may contribute to oxidative stress generation that promotes neoplastic transformation and/or maintenance of the malignant phenotype. In looking at the correlation between MnSOD expression and cancer progression, mutations within the MnSOD gene and its regulatory sequence have been observed in several types of human cancers [52, 53]. However, antioxidants can suppress carcinogenesis, particularly during the promotion phase. In addition, our laboratory as well as others has shown that overexpression of MnSOD reduces tumor multiplicity, incidence, and metastatic ability in various *in vitro* and *in vivo* models [54–57].

## 4. The Tumor Suppressor p53

p53 is a well-characterized transcription factor known to induce its tumor suppressor activity by activating genes known to play a role in cell cycle arrest, such as p21^CIP1^ and *GADD45*. These genes, once activated, arrest the cell cycle to allow for adequate DNA repair to restore normal cell proliferation. However, if the cell becomes overwhelmed by the stressor or the DNA damage cannot be repaired, p53 can ultimately induce apoptosis. The tumor suppressive activities of p53 can also be defined by the induction senescence. Senescence is characterized by irreversible loss of proliferative potential, acquisition of characteristic morphology, and expression of specific biomarkers such as senescence-associated *β*-galactosidase [58]. Nevertheless, how p53 regulates senescence is often contradictory and dependent on ROS generation. p53 can mediate cellular senescence via the transactivation of p21^CIP1^. Nonetheless, emerging evidence suggests ROS as a common mediator of senescence with the involvement of superoxide dismutase and p53. Blander et al. reported that RNAi-mediated knockdown of SOD1 in primary human fibroblasts induced cellular senescence mediated by p53. However, senescence was not induced in p53-deficient human fibroblasts [59]. Furthermore overexpression of MnSOD induced growth arrest in the human colorectal cancer cell line HCT116 and increased senescence which required the induction of p53 [60]. On the contrary, p53 can suppress senescence through the inhibition of the mTOR pathway via multiple mechanisms [61–63]. Nevertheless, this diverse biological spectrum of p53 regulation of cellular function remains complex and is dependent on the source of activation and cell type. 

**Figure 1:**
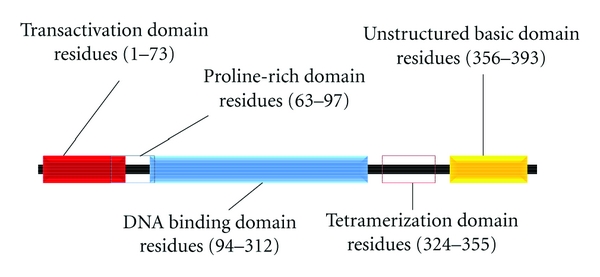
p53 Multifunctional domains. The p53 monomer consists of various multifunctional domains including the N-terminal transactivation domain (residues 1–73), a proline-rich region (residues 63–97), the highly conserved DNA-binding core domain (residues 94–312), a tetramerization domain located within the C-terminus (residues 324–355), and an unstructured basic domain (residues 360–393).

 There are various sources of p53 activators, which include nucleotide depletion, hypoxia, ultraviolet radiation, ionizing radiation, as well as many chemotherapeutic drugs can act as activators of p53 (i.e., Doxorubicin). In normal cells, p53 remains at a low level and is under strict control by its negative regulator Mdm2. p53 induces autoregulation via Mdm2. As a transcription factor, p53 can bind to the promoter region of the *mdm2* gene to promote transcription of Mdm2 mRNA [64, 65]. Following proper translation into a functional protein, Mdm2 acts as an E3 ligase during p53 activation. Mdm2 can polyubiquitinate p53 leading to proteasomal degradation [66]. However, Mdm2 can also monoubiquinate p53 leading to intracellular trafficking [67]. The decisive role of p53 to induce cell cycle arrest, senescence, or apoptosis involves intricate posttranslational, as well as, transcription-dependent and transcription-independent mechanisms. The tumor suppressor p53 is a well-characterized transcription factor known to induce the transactivation of proapoptotic genes such as Bax, Puma, Noxa, Bid and represses the transcription of anti-apoptotic genes such as Bcl-2, Bcl-xL, and survivin [68, 69]. Nevertheless, p53 can induce apoptosis independent of its transcriptional activity. Many of the transcription-independent mechanisms of p53 were discovered through the use of inhibitors of transcription/translation, as well as p53 truncated mutants with altered subcellular localization, DNA binding, and cofactor recruitment. The p53 monomer consists of various multifunctional domains including the N-terminal transactivation domain (residues 1–73), a proline-rich region (residues 63–97), the highly conserved DNA-binding core domain (residues 94–312), a tetramerization domain located within the C-terminus (residues 324–355), and an unstructured basic domain (residues 360–393) [70] (Figure 1). There are multiple polymorphisms that occur within the *TP3* gene that may enhance or alter p53 functionality. Dumont et al. discovered functional differences in polymorphic variants that enhanced p53-mediated apoptosis independent of its transactivation abilities [71]. A common sequence polymorphism that occurs within the proline-rich domain encoding arginine at position 72 exhibited a fivefold increase in inducing apoptosis compared to the common proline (Pro72) variant. These results suggested two mechanisms of Arg 72 apoptotic enhancement: (1) increased mitochondrial localization; (2) enhanced binding of the Arg 72 variant to the negative p53 regulator E3 ligase, Mdm2. Although increased binding to Mdm2 did not augment p53 degradation, it was suggested that the altered confirmation of the p53 Arg 72 variant enhanced the binding ability and facilitated greater nuclear export [71]. This suggests the importance of understanding the regulation of structure-activity relationships in polymorphic forms of p53 in transcription-independent apoptosis.

During p53-mediated apoptosis, a distinct cytoplasmic pool of p53 translocates to the mitochondria. To promote mitochondrial translocation, the E3 ligase, Mdm2 monoubiquitinates p53 [72]. Since the p53 protein lacks a mitochondrial localization sequence, p53 interacts with Bcl-2 family proteins via Bcl-2 homology (BH) domains. The presence of the BH domain allows proteins to regulate and interact with other Bcl-2 members that consist of multiple BH domains [73]. Once p53 arrives at the mitochondrial outer membrane, p53 binds to Bak inducing a conformational change and Bak homo-oligomerization that results in mitochondrial outer membrane permeabilization (MOMP). MOMP allows for the release of pro-apoptotic signaling molecules from the outer and inner mitochondrial membranes into the cytosol triggering the intrinsic apoptotic signaling cascade. ROS generation has been suggested as an alternative p53 apoptotic target independent of cytochrome *c* release. Li et al. found that ROS generation regulated the mitochondrial membrane potential (Δ*ψ*), which was found to be a key constituent in the induction of p53-mediated apoptosis [74]. Interestingly, during ROS generation, apoptosis occurred in the absence of Bax mitochondrial translocation, Bid activation, as well as cytochrome *c* release. Several studies have suggested that the downstream effects of p53-mediated apoptosis are regulated by Bax expression. It has been shown that the introduction of recombinant Bax protein into isolated mitochondria induced cytochrome *c* release. The ability of Bax to initiate pore formation in synthetic membranes has been shown to regulate cytochrome *c* release resulting in the induction of apoptosis [75, 76]. However, discrepancies exist with *in vivo* studies showing Bax being localized in the cytosol, rather than within the mitochondrial membrane at physiological conditions [77].

Herein, we show how p53 has been shown to play a dual role in early-versus late-stage cancer progression. During the process of carcinogenesis, mutations can occur both upstream and downstream of p53 activation. For example, loss of upstream activators of p53, for example, ATM and Chk2, can prevent p53 activation, contributing to unregulated cell cycling and promoting tumorigenesis [78]. In addition, mutations within the p53 protein can alter necessary structure conformational changes and DNA binding properties needed for efficient p53 activation. Lastly, many of these mutations lead to loss of downstream genes such as Bax or NOXA which are pro-apoptotic and necessary for regulation of cellular proliferation and death signaling. 

The process of tumor formation is a multistage process that involves both the activation of protooncogenes, and the inactivation of tumor suppressor genes, such as PTEN and p53. The multistage carcinogenesis paradigm consists of three well-characterized stages: initiation, promotion, and progression. During the initiation stage, there is the induction of mutations within critical target genes of stem cells, for example, H-ras; however in the skin carcinogenesis model, the epidermal layer remains phenotypically normal. During the tumor promotion stage, a noncarcinogenic agent such as a phorbol ester can be used to induce the clonal expansion of the initiated stem cells through epigenetic mechanisms. This stage is often used by investigators to identify potential therapeutic targets due to its reversibility. During the tumor progression stage, malignancy takes place, being characterized by enhanced invasiveness via the activation of proteases, and metastasizes via tumor cells entering into the lymphatics and loss of tumor suppressor activity (e.g., p53).

**Figure 2:**
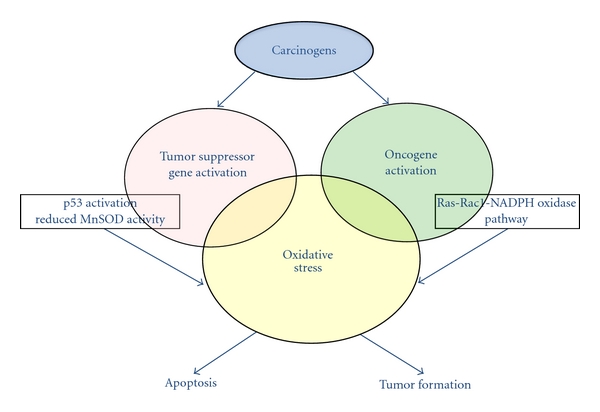
Mechanisms of carcinogens in early stage carcinogenesis. During the early stages of tumor promotion both oncogenes and tumor suppressor genes are activated, resulting in increased cell proliferation being accompanied by increased cell death.

The two-stage skin carcinogenesis mouse model has been well characterized and used in numerous studies to screen anti cancer agents. An initiator, such as dimethylbenz[a]anthracene (DMBA), is applied to the skin to initiate DNA damage within skin cells. Following DMBA treatment, a tumor promoter such as 12-*O*-tetradecanoylphorbol-13-acetate (TPA) is applied topically to the same area repeatedly for the duration of the study to promote the clonal expansion of mutated cells during the promotion stage. Interestingly, during the early stages of DMBA/TPA-mediated tumor promotion both oncogenes and tumor suppressor genes are activated, resulting in increased cell proliferation being accompanied by increased cell death [79] (Figure 2). Both processes exist throughout skin tumor formation. Not surprising, these two opposing events are closely related. 

Many of the tumor-promoting mechanisms utilized by phorbol esters are directly linked to the involvement of cell surface membranes [80, 81]. TPA can mediate its pleiotropic actions through intercalating into the cellular membrane and inducing the activation of the Ca^2+^-activated phospholipid-dependent protein kinase, protein kinase C (PKC) both *in vitro *and* in vivo*. TPA can directly activate PKC via molecular mimicry by substituting for diacylglycerol, the endogenous substrate, increasing the affinity of PKC for Ca^2+^ which leads to the activation of numerous downstream signaling pathways involved in a variety of cellular functions including proliferation and neoplastic transformation [82]. In addition, it is known that a direct correlation exists between phorbol ester-mediated tumor promotion and enzymatic activation of PKC [82, 83]. The PKC family consists of various highly conserved serine/threonine kinases. PKCs are involved in numerous cellular processes including cell differentiation, tumorigenesis, cell death, aging, and neurodegeneration [84]; however the induction of the signaling pathway is determined by the intracellular redox status and the isoform that is activated. The PKC family consists of a myriad of isoforms that have been divided into three classes: (a) classical or conventional PKCs (cPKC: *α*, *β*I, *β*II, and *γ*); (b) novel PKCs (nPKC: *δ*, *ε*, *η*, and *θ*); (c) the atypical PKCs (aPKC: *λ*, *ι*, and *ζ*) which are classified based on sensitivity to Ca^2+^ and diacylglycerol (DAG) [84]. In various types of cancers PKC*ε* has been shown to be upregulated while PKC*α* and PKC*δ* are downregulated. Interestingly, TPA activates the PKC*ε* isoform in mouse skin tissues [85]. Furthermore, overexpression of PKC*ε* has been shown to enhance the formation of skin carcinomas [86]. Moreover, TPA treatment leads to the concomitant activation of the redox-sensitive transcription factor activator protein-1 (AP-1) [85]. The AP-1 complex consists of both Jun and Fos oncoproteins. There are 3 jun isoforms (c-jun, jun-B, and jun-D) and 4 fos family members (c-fos, fra-1, fra-2, and fos-B) [87] whose activation is modulated by oxidants such as superoxide and hydrogen peroxide, while DNA binding activities are modulated by the intracellular redox status [88–90]. Kiningham and Clair reported a reduction in tumorigenicity and AP-1 DNA binding activity following overexpression of MnSOD in transfected fibrosarcoma cells [91]. Furthermore, the protein expression of Bcl-xl, an antiapoptotic AP-1 target gene, was decreased, as well. In addition, PKC*ε* activation was reduced in MnSOD transgenic mice treated with DMBA/TPA compared to their nontransgenic counterparts [85]. These results suggest a mechanistic linkage between MnSOD expression, mitogenic activation, and AP-1 binding activity.

## 5. MnSOD-p53 Mitochondrial Interaction

Another activated signaling pathway that has been defined following DMBA/TPA treatment is the Ras-Rac1-NADPH oxidase pathway, which leads to p53 mitochondrial translocation and apoptosis [92]. NADPH oxidase forms a stable heterodimer with the membrane protein p22^phox^, which serves as a docking site for the SH3 domain-containing regulatory proteins p47^phox^, p67^phox^, and p40^phox^. Upon TPA treatment, Rac, a small GTPase, binds to p67^phox^ which induces NADPH oxidase activation [11] and superoxide production. Mitochondrial p53 has been shown to interact with MnSOD, resulting in decreased enzymatic activity and promoting oxidative stress propagation [93].

**Figure 3:**
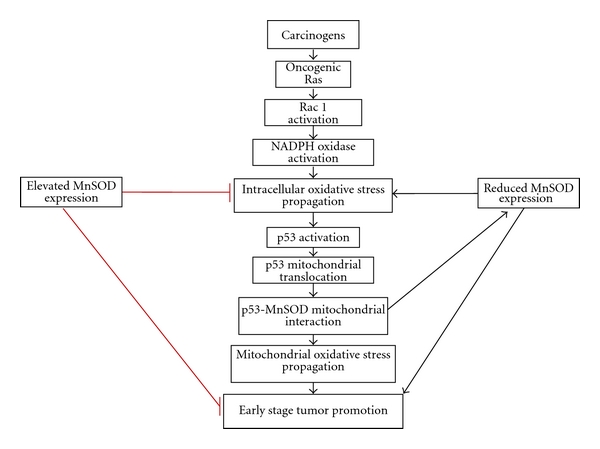
Mechanism involving the p53-MnSOD interaction during the early stage of tumor promotion. Following exposure to a carcinogen the Ras-Rac1-NADPH oxidase pathway is activated, which leads to p53 mitochondrial translocation. Mitochondrial p53 has been shown to interact with MnSOD, resulting in decreased enzymatic activity and promoting oxidative stress propagation contributing to the early stage of skin tumorigenicity. Elevated levels of MnSOD reduced oxidative stress propagation, suppressed p53 mitochondrial translocation, and decreased downstream skin tumor formation. Reduced levels of MnSOD have been shown to contribute to oxidative stress propagation and promote early-stage skin tumorigenicity.

The primary role of MnSOD is to protect mitochondria from oxidative damage. In 2005, Zhao et al. found that TPA treatment, both *in vitro* and *in vivo*, can induce p53 mitochondrial translocation [93]. In addition, p53 not only came in contact with the outer mitochondrial membrane but was able to localize to the mitochondrial matrix. Interestingly, following p53 mitochondrial translocation and matrix localization, p53 interacted with the mitochondrial antioxidant enzyme MnSOD that resulted in a reduction in MnSOD activity and propagation of oxidative stress [93]. However, the question remains: does mitochondrial p53 contribute to or suppress tumor promotion during the early stages of skin carcinogenesis? We addressed this question by utilizing the JB6 mouse skin epidermal cells. JB6 cells were originally derived from primary BALB/c mouse epidermal cell culture [94]. Through nonselective cloning, it was discovered that clonal variants existed within the JB6 cell lineage that were either stably sensitive (P+) or resistant (P−) to tumor promoter-induced neoplastic transformation [95–97]. In addition, JB6 cells remain the only well-characterized skin keratinocytes for studying tumor promotion and screening anti-cancer agents. In 2010, we utilized the JB6 P+ and P− clonal variants to determine if a relationship existed between tumor promotion and early-stage TPA-induced p53 activation [98]. Surprisingly, we found that p53 was only induced in promotion-sensitive P+ cells and not promotion resistant (P−) cells, therefore suggesting that p53 expression is highly associated with early stage tumor promotion. We then assessed Bax protein expression levels, as a marker for p53 transcriptional activity, and found that Bax expression is only induced in JB6 P+ cells and not P− cells, suggesting that p53 expression, as well as transcriptional activity, is highly associated with early-stage tumor promotion following TPA treatment. MnSOD expression was also measured in both JB6 P+ and P− cells and was found to be highly expressed in promotion-resistant P− cells compared to promotable P+ cells. TPA-mediated ROS generation was measured in P+ and P− cells (unpublished data), and promotion resistant cells contained significantly lower levels of ROS following TPA treatment when compared to their promotable counterparts. It is known that reduced MnSOD expression contributes to increased DNA damage, cancer incidence, and radical-caused diseases [99, 100]. Consistent with that, an increase of several markers of oxidative damage such as 4-HNE, 8-oxoDG, and lipid peroxidation has been seen in both *in vitro *and *in vivo* studies following TPA treatment [57, 85, 101, 102], suggesting the involvement of oxidative stress in the promotion of tumorigenesis. These results imply the importance of redox regulation in modulating cellular functions during the early stage of tumor promotion. We questioned whether the ROS generated from the MnSOD-p53 mitochondrial interaction was sufficient to promote tumorigenicity. Therefore, we utilized promotion-resistant JB6 P− cells that exhibited no p53 protein expression or transactivation following TPA treatment to address this question. Interestingly, we found that when JB6 promotion-resistant cells were transfected with wild-type p53, these cells were able to transform and form colonies in soft agar, in comparison to their control counterparts [98]. These results suggest a dual role of p53-mediated ROS generation during the early stages of skin carcinogenesis and how the presence of p53 is necessary for tumor promotion in skin (Figure 3). 

The contradictory role of p53 in promoting cell survival or death is the result of the ability to regulate the expression of both pro- and antioxidant genes. For example, p53 can promote the generation of ROS through the induction of genes involved in mitochondrial injury and cell death which include Bax, Puma, and p66^SHC^ and ROS-generating enzymes such as quinine oxidoreductase (NQO1) and proline oxidase [103]. However, p53 can upregulate the expression of various antioxidant enzymes to modulate ROS levels and promote cell survival such as aldehyde dehydrogenase 4 and mammalian sestrin homologues that encode peroxiredoxins and GPX1, which are major enzymatic removers of peroxide [103].

Dhar et al. suggested that p53 possessed “bidirectional” regulation of the antioxidant MnSOD gene. Previous reports suggest the presence of a p53 binding region at 328 bp and 2032 bp upstream of the transcriptional start site of the MnSOD gene [104, 105]. Others suggest that p53 represses MnSOD gene expression by interfering with transcription initiation [106], inhibiting gene activators at the promoter level by forming an inhibitory complex suppressing gene transcription [107] and protein-protein interactions [108]. Nevertheless, p53 can induce the gene expression of MnSOD [104]. p53-mediated MnSOD expression is regulated in conjunction with other cell proliferative transcription factors such as NF-*κ*B. Kiningham and Clair demonstrated the presence of an NF-*κ*B binding site within the intronic enhancer element of the MnSOD gene [91]. It was later shown that mutation of the NF-*κ*B site within the enhancer element abrogated p53 induced MnSOD gene transcription. In addition, knockdown of p65 via siRNA reduced MnSOD gene transcription via p53 as well. Overall the effects of p53 on MnSOD gene expression have been suggested to be concentration dependent, with low concentrations of p53 increasing MnSOD expression via corroborative NF-*κ*B binding promoting cell survival and high concentrations of p53 suppressing MnSOD expression by interfering with important transcriptional binding elements such as SP1.

## 6. Clinical Implications of the MnSOD-p53 Interaction

p53 is mutated in 50% of human cancers. However, the remaining human tumors contain wild-type p53 with defects in the downstream mediated p53-signaling pathways. This, in turn, provides novel areas of discovery in stabilization and restoration of wild-type p53 activity. Currently, many drug companies are focused on utilizing p53 interactions as targets for pharmacological intervention [78]. There are various protein-protein interactions that occur within the cell that positively or negatively regulate p53 expression and function. For example, Mdm2 is an E3 ligase of p53 that polyubiquitinates p53, priming the tumor suppressor for proteasomal degradation. Many have found that, by blocking this interaction through peptides or transcriptional inhibitors, longer durations of p53 activation have resulted. Some of the therapeutic strategies that are currently being utilized are peptides that increase p53 activation through inhibition of Mdm2 function [109]. Three-dimensional structural models [110] of the hdm2-p53 interaction along with biochemical data [111, 112] have identified three residues that are important to this interaction, Phe19, Trp23, and Leu26 [111, 112]. From this data, an 8-mer peptide was generated [113] and showed promising results in inducing apoptosis in tumor cells that overexpressed hdm2 [112]. However, these conditions were difficult to optimize with a smaller molecule therefore causing this peptide to be therapeutically inefficient. Also nutlins have been utilized to disrupt the mdm2-p53 interaction resulting in reactivation of the p53 response [114, 115]. Others have used antisense and transcription inhibitors to prevent the expression of Mdm2 [116]. 

Gene replacement therapy is another therapeutic modality that has been explored in treating tumors lacking or containing mutant p53. This technique utilizes adenoviruses, as well as retroviruses to achieve high expression of p53 in tumor cells. Promising results have been seen with retroviral vectors in patients with nonsmall cell lung cancers [117]. On the contrary, although we have seen the enhancement of tumorigenicity in our *in vitro* p53 transfection studies [98], we have not tested stably transfected cells in *in vivo* xenograft mouse models, nor have we tried other tissue types. Therefore, the reintroduction of the p53 gene into tumors may have contradictory outcomes depending on the cell type and tissue microenvironment. This concern has echoed through various studies, persuading investigators to opt to combine gene therapy with chemotherapy and radiotherapy [118–121].

For decades, it has been shown that p53 functions only as a tumor suppressor. In addition, p53-mediated ROS generation has been limited to the induction of apoptosis. Currently, the ability of wild-type p53 to contribute to tumor promotion has received considerable attention. We have shown that the p53-MnSOD interaction contributes to the early stage of tumor promotion. In addition, it has been consistently shown that MnSOD activity is altered in human tumors. Therefore, designing diagnostic tools to assess MnSOD activity, as well as p53 activation, can be used to effectively design individualized treatments for cancer patients. For example, following chemotherapeutic treatment, patients that have higher levels of p53 expression and exhibit lower levels of MnSOD can receive an SOD mimetic that can upregulate MnSOD or synthetic compounds that can downregulate p53 activity to decrease ROS-mediated apoptosis and potential relapse within these patients.

Gene therapy has also been utilized to modulate MnSOD activity during cancer progression. Overexpression of MnSOD through gene therapy introducing genetically engineered DNA/liposomes containing the human MnSOD transgene into preclinical and clinical models has been shown to be protective in normal tissues against ionizing irradiation. The final product (VLTS-582) is a DNA/liposome formulation that consists of a double-stranded DNA bacterial plasmid containing human MnSOD cDNA in conjunction with two lipids {cholesterol and DOTIM (1-[2-[9-(2)-octadecenoyloxy]]-2-[8-(2)-heptadecenyl]-3-[hydroxyethyl] imidazolinium chloride)} [122]. Recent studies suggest that this formulation has been successful in murine models and has been administered orally to patients concurrently with a weekly chemotherapy regime exhibiting no dose-limiting toxicities. Although proven therapeutically efficacious, more studies are needed to improve (1) delivery of the transgene to the targeted tissue; (2) reducing rapid elimination of the transgene; (3) control of the expression of the transgene within targeted tissues.

On the other hand, a topical application of an SOD mimetic has also been described [123]. The Mn (III) porphyrin Mn^III^ TE-2-Pyp^5+^ possesses highly potent SOD activity as facilitated by the redox properties of the metal center and the positive charge to the ortho-N-ethylpyridyl nitrogens [124]. Mn^III^ TE-2-Pyp^5+^ has been proven effective *in vitro* and in various human diseases such as stroke [125, 126], diabetes [127, 128], and cancer and radiation-related treatment [129–132]. In preclinical animal models, topical application of Mn^III^ TE-2-Pyp^5+^ was shown to reduce levels of oxidative damage and reduced cell proliferation without interfering with p53-mediated apoptosis when applied prior to TPA treatment [129]. These data support the concept that overexpression of MnSOD when applied in conjunction with standard chemotherapeutics or during the tumor promotion stage is protective in both preclinical and clinical models.

Nevertheless, both p53 and MnSOD have been shown to posses reduced activity and/or mutated in most human diseases including cancer. Therefore, more therapeutic quests are needed to detect and restore both MnSOD and wild-type p53 activity. However, future therapeutic optimization strategies should have minimal nonspecific drug-related toxicities and be based on the stage of cancer progression which may reveal a therapeutic window for treatment intervention.

## 7. Concluding Remarks

In summary, reactive oxygen species have been implicated in the pathogenesis of various hyperproliferative and inflammatory diseases [133]. In addition, the tumor suppressor p53 has been shown to be activated during the early stage of skin carcinogenesis and contributed to the propagation of oxidative stress. Recent studies demonstrate a novel role of mitochondrial p53 activation. Once in the mitochondria, p53 physically interacts with MnSOD. As a result, this interaction reduces the free radical scavenging abilities of MnSOD, promoting enhanced ROS generation which has been shown to act as a tumorigenic stimulus during cancer progression. This suggests that wild-type p53 may play a direct role in promoting oxidative stress and contributing to the ROS-mediated tumor-proliferative stimuli. In addition, others have shown that mutant p53 can, in fact, translocate to the mitochondria and interact with MnSOD [134]. However, Lontz et al. observed following doxorubicin treatment of lymphoma cell lines with varying wt or mutant p53 levels, mitochondrial function, as evidenced by Complex I/II activities, was only compromised in lymphoma cells expressing wild-type and not mutant p53 [134]. Therefore, the continuation of deciphering mechanistic differences in tumors containing wild-type or mutant p53 can lead to the development of therapeutic p53-mediated interventions and a clearer understanding of chemoresistance in both wild-type and mutant p53 human tumors.

Several studies have suggested that MnSOD may play a primary protective role against tissue injury. MnSOD has been found to be depleted in a variety of tumor cells, as well as *in vitro* transformed cell lines, suggesting that MnSOD may act as a novel tumor suppressor, protecting cells from oxidant-induced carcinogenesis [135]. Nevertheless, overexpression of MnSOD decreases the pathogenesis of human diseases such as cancer. Consistent with that, accumulating evidence suggests that a number of antioxidants or drugs with antioxidant properties can reduce mediators of tumor promotion [136]. Clair et al. showed that transfecting mouse 10T 1/2 cells with human MnSOD cDNA promoted differentiation with 5-azacytidine treatment and protected against neoplastic transformation [137]. In addition, transfecting human MnSOD cDNA into MCF-7 breast cancer cells and UACC-903melanoma cells suppressed their malignant phenotype and suppressed growth in nude mice [54, 138]. We have shown that the cumulative induction of endogenous antioxidant enzymes (i.e., catalase, total SOD and MnSOD) is efficient in reducing tumor incidence and multiplicity [57]. In addition, the induction of endogenous antioxidant enzymes via dietary administration can suppress p53 mitochondrial translocation [98]. TPA can induce p53 mitochondrial translocation; however, this phorbol ester also decreases the mitochondrial membrane potential, as well as mitochondrial complex activities and respiration. Other studies have shown that MnSOD overexpression in mice protects complex I from adriamycin-induced deactivation in cardiac tissue [139]. These results suggest that antioxidant expression protects against fluctuations in mitochondrial functions which suppress p53 mitochondrial translocation, p53-mediated ROS, and both downstream apoptotic and cell proliferation signaling pathways. On the contrary, Connor et al. suggest that overexpression of MnSOD in HT-1080 fibrosarcoma cells and 253J bladder tumor cells enhanced the migatory ability and invasiveness of tumor cells, through the upregulation of matrix metalloproteinases [140]. Although some tumors express higher levels of MnSOD, the downstream effects of enhanced antioxidant expression are dependent on the tumor type and susceptibility to oxidative damage, underlying oncogenic mutations and the stage of disease progression [140]. Nevertheless, these investigators stressed the need of refined regulation of H_2_O_2_ production. Therefore the question remains, are the effects of the p53-MnSOD interaction protumorigenic or anti-tumorigenic? To definitively answer this question further investigation of this interaction is needed. However, there are several factors that must be considered in determining the fate of the p53-MnSOD interaction, which include the stage of disease progression as well as tumor microenvironment. It has been shown that p53 activation is required in tumor promotion and can mediate ROS generation. However, the duration of enhanced ROS generation, severity of oxidative damage, and the status of the cellular antioxidant capacity can all contribute to the proliferative/apoptotic switch that occurs during the response to cellular stress. Overall, further studies are needed to clearly assess the status of MnSOD during the various stages of carcinogenesis to enhance the efficacy of standard treatment regimens currently being used.

Consistent with that, defining the downstream effects of the p53-MnSOD complex formation can expand our knowledge of the molecular mechanisms that contribute to the early stage of tumorigenesis and how they may be altered during cancer progression. With further knowledge, modulators of MnSOD, p53 and their associated regulators can be therapeutically useful in the treatment of cancer and various stages of tumor progression.
